# Cloning of a Conserved Receptor-Like Protein Kinase Gene and Its Use as a Functional Marker for Homoeologous Group-2 Chromosomes of the *Triticeae* Species

**DOI:** 10.1371/journal.pone.0049718

**Published:** 2012-12-13

**Authors:** Bi Qin, Tingting Chen, Aizhong Cao, Haiyan Wang, Liping Xing, Hongqing Ling, Daowen Wang, Chunmei Yu, Jin Xiao, Jianhui Ji, Xueluan Chen, Peidu Chen, Dajun Liu, Xiue Wang

**Affiliations:** 1 The State Key Laboratory of Crop Genetics and Germplasm Enhancement, Cytogenetics Institute, Nanjing Agricultural University, Nanjing, Jiangsu, China; 2 The State Key Laboratory of Plant Cell and Chromosome Engineering, National Center for Plant Gene Research, Institute of Genetics and Developmental Biology, Chinese Academy of Sciences, Beijing, China; 3 Key Laboratory of Biology and Genetic Resources of Rubber Tree, Ministry of Agriculture, Rubber Research Institute, Chinese Academy of Tropical Agricultural Sciences, Danzhou, Hainan, China; National Rice Research Center, United States of America

## Abstract

Receptor-like kinases (RLKs) play broad biological roles in plants. We report on a conserved receptor-like protein kinase (*RPK*) gene from wheat and other *Triticeae* species. The *TaRPK1* was isolated from the *Triticum aestivum* cv. Prins - *Triticum timopheevii* introgression line IGVI-465 carrying the powdery mildew resistance gene *Pm6*. The *TaRPK1* was mapped to homoeologous chromosomes 2A (*TaRPK1-2A*), 2D (*TaRPK1-2D*) and the *Pm6*-carrier chromosome 2G (*TaRPK1-2G*) of IGVI-465. Under the tested conditions, only the *TaRPK1-2G* allele was actively transcribed, producing two distinct transcripts via alternative splicing. The predicted 424-amino acid protein of TaRPK1-2G contained a signal peptide, a transmembrane domain and an intracellular serine/threonine kinase domain, but lacked a typical extracellular domain. The expression of *TaRPK1-2G* gene was up-regulated upon the infection by *Blumeria graminis* f.sp. *tritici* (*Bgt*) and treatment with methyl jasmonate (MeJA), but down-regulated in response to treatments of SA and ABA. Over-expression of *TaRPK1-2G* in the powdery mildew susceptible wheat variety Prins by a transient expression assay showed that it slightly reduced the haustorium index of the infected *Bgt*. These data indicated that *TaRPK1-2G* participated in the defense response to *Bgt* infection and in the JA signaling pathway. Phylogenetic analysis indicated that *TaRPK1-2G* was highly conserved among plant species, and the amino acid sequence similarity of TaRPK1-2G among grass species was more than 86%. Based on its conservation, the *RPK* gene-based STS primers were designed, and used to amplify the *RPK* orthologs from the homoeologous group-2 chromosomes of all the tested *Triticeae* species, such as chromosome 2G of *T. timopheevii*, 2R of *Secale cereale*, 2H of *Hordeum vulgare*, 2S of *Aegilops speltoides*, 2S^l^ of *Ae. longissima*, 2M^g^ of *Ae. geniculata*, 2S^p^ and 2U^p^ of *Ae. peregrina*. The developed STS markers serve as conserved functional markers for the identification of homoeologous group-2 chromosomes of the *Triticeae* species.

## Introduction

Receptor protein kinases (RPKs) play essential roles in the signal perception in animals in response to various growth factors and hormones [1]. These receptors generally have an extracellular domain, a single transmembrane domain, and an intracellular catalytic kinase domain. Ligands bound by the extracellular domain stimulates receptor autophosphorylation on tyrosine residues within the cytoplasmic protein kinase domain. Then the binding of the ligand to the extracellular domain causes receptor dimerization thereby activating the cytoplasmic kinase domain by intermolecular phosphorylation and transduction of the signal to the downstream effectors [2]. Based on the primary structure, plants also have a large gene family named as receptor-like kinases (RLKs) similar to the animal's RPKs, however, the auto-phosphorylation in plant RLKs is mostly specific to the serine and/or threonine [3]. Plant RLKs include receptor kinases and receptor-like cytoplasmic kinases (RLCKs) with no typical signal sequence or transmembrane domain, which have been implicated in the perception and transduction of extracellular signals into the cell [4]. The RLKs are usually encoded by hundreds of genes in plant genomes, for example, *Arabidopsis* has more than 600 predicted RLKs representing nearly 2.5% of all the coding genes, and rice (*Oryza sativa*) has nearly twice as many RLKs members as *Arabidopsis* does [5]. Due to the large gene family, RLKs vary greatly for both their domain organization and the extracellular domains, and the RLK family can be divided into more than 40 sub-families based on their distinct extracellular domains [4]. The diversity in the ligand binding domain endows the RLKs a wide range of biological function, such as growth and development, responses to biotic and abiotic stresses, and nodulation and rhizobial symbiosis [5].

Plant RLKs have been implicated their roles in diverse signaling pathways. For example, *Pto* provides resistance against *Pseudomonas syringae* in tomato [6], *SLG* of *Brassica oleracea* may be required for self-incompatibility in the recognition between pollen and stigma [7], [8], and the rice *Xa21* confers broad spectrum resistance to *Xanthomonas oryzae* pv. *oryzae* [9]. Several RLKs have also been identified in wheat. The WLRK (wheat leaf rust kinase) gene family, which is located at the *Lr10* locus and conserved in wheat and related species, confers resistance to leaf rust disease [10]–[12]. Three receptor-like kinase genes (*TaRLK-R1*, *2* and *3*) were found to participate in the wheat hypersensitive response to the stripe rust fungus [13]. A recent research in our lab showed that a putative serine and threonine protein kinase gene, *Stpk-V*, which located to the *Pm21* locus from *Haynaldia villosa*, conferred broad-spectrum powdery mildew resistance in wheat [14]. However, compared to the large population of the RLK genes in wheat, information on their biological role is still very limited. Thus, the identification of more wheat RLK genes is critical for better understanding of their biological roles in wheat.

In our previous studies, a barley RFLP probe BCD135 was found to be closely linked with the powdery mildew resistance gene *Pm6*, which has been introduced from the tetraploid wheat *T. timopheevii* into the hexaploid common wheat. The sequence of BCD135 was highly conserved among several species including barley, wheat, rye, and rice [15]. At the genome region of BCD135 in barley and rice, there were two conserved genes, one was a putative receptor-like protein kinase gene (*HvRPK*), and the other had no putative function [16]. Based on the sequence of the second gene in barley, Ji et al. developed two STS markers closely linked to the gene *Pm6* in common wheat [17]. In the present study, the conserved *TaRPK1* gene in this region was explored in grass species based on the barley *HvRPK*, and a functional marker was developed for the identification of the homoeologous group-2 chromosomes of *Triticeae* species. The *TaRPK1* genes belonging to this conserved family were further cloned and characterized from hexaploid wheat, and the putative biological function of *TaRPK1-2G* was investigated.

## Materials and Methods

### Plant materials

Different *Triticeae* species with various genome constitution including *Secale cereale* L. cv. ‘BLANCO’ (2n = 2x = 14, RR), *Hordeum vulgare* L. cv. ‘BETZES’ (2n = 2x = 14, HH), *Aegilops speltoides* Tausch (2n = 2x = 14, SS), *Ae. longissima* Schw. et Musch (2n = 2x = 14, S^l^S^l^), *Ae. geniculata* Roth (2n = 2x = 14, M^g^M^g^), and *Ae. peregrina* (Hack.) Maire & Weiller (2n = 4x = 28, S^p^S^p^U^p^U^p^]), and their genetic stocks, i.e. addition lines with the alien chromosomes added in the background of wheat variety ‘Chinese spring’ (CS) (*Triticum aestivum* L., 2n = 6x = 42, AABBDD) were introduced from Wheat Genetics & Genomic Resources Center (WGGRC), Kansas State University, USA (Table 1). The Swedish common wheat variety Prins, which is susceptible to powdery mildew, one *T. timopheevii* (2n = 4x = 28, AAGG) accession with the powdery mildew resistance gene *Pm6*, two *Pm6*-carrying *T. aestivum*-*T. timopheevii* introgression lines (IGVI-465 [FAO 163b/7*Prins] and IGVI-466 [Kenya Lemphi 50-13596/7*Prins]) were kindly provided by Dr. J. MacKey, Swedish Agricultural University, Uppsala, Sweden, and were used for gene cloning. A set of CS nulli-tetrasomic lines of homoeologous group-2 were also provided by the WGGRC and were used for mapping of wheat *RPK* genes.

**Table 1:** Plant materials introduced from the WGGRC, KSU, USA.

Accession number	Materials	Genome or Chromosome constitution
TA9001	*H. vulgare* L. cv. ‘BETZES’	HH
TA3698	CS- BETZES DA 2H	21″+t″[2H arm unknown]
TA3699	CS- BETZES DA 3H	21″+1″[3H]
TA3700	CS- BETZES DA 4H	21″+1″[4H]
TA3701	CS- BETZES DA 5H	21″+1″[5H]
TA3702	CS- BETZES DA 6H	21″+1″[6H]
TA7591	CS-*H. chilense* DA 7H^∧^ch^∧^	21″+1″[7H^∧^ch^∧^]
TA9020	*Secale* L.cv.‘BLANCO’	RR
TA7501	CS- BLANCO DA 1R	21″+1″[1R]
TA7502	CS- BLANCO DA 2R	21″+1″[2R]
TA7503	CS- BLANCO DA 3R	21″+1″[3R]
TA7505	CS- BLANCO DA 4R	21″+1″[4R]
TA7506	CS- BLANCO DA 5R	21″+1″[5R]
TA7507	CS- BLANCO DA 6R	21″+1″[6R]
TA7508	CS- BLANCO DA 7R	21″+1″[7R]
TA1910	*Ae. longissima*	S^l^S^l^
TA7543	CS- *Ae. longissima* DA 1S^∧^l^∧^#3	21″+1″[1S^∧^l^∧^#3]
TA7544	CS- *Ae. longissima* DA 2S^∧^l^∧^#3	21″+1″[2S^∧^l^∧^#3]
TA7545	CS- *Ae. longissima* DA 3S^∧^l^∧^#3	21″+1″[3S^∧^l^∧^#3]
TA7546	CS- *Ae. longissima* DA 4S^∧^l^∧^#3	21″+1″[4S^∧^l^∧^#3]
TA7547	CS- *Ae. longissima* DA 5S^∧^l^∧^#3	21″+1″[5S^∧^l^∧^#3]
TA7548	CS- *Ae. longissima* DA 6S^∧^l^∧^#3	21″+1″[6S^∧^l^∧^#3]
TA7550	CS- *Ae. longissima* DA 2S^∧^l^∧^#4	21″+1″[2S^∧^l^∧^#4]
TA3579	CS-*Ae. longissima* DA 7/4S^∧^l^∧^#2	21″+1″[7/4S^∧^l^∧^#2]
TA2899	*Ae. geniculata*	M^g^M^g^
TA7655	CS- *Ae. geniculata* DA 1M^∧^g^∧^#1	21″+1″[1M^∧^g^∧^#1]
TA7656	CS- *Ae. geniculata* DA 2M^∧^g^∧^#1	21″+1″[2M^∧^g^∧^#1]
TA7657	CS- *Ae. geniculata* DA 3M^∧^g^∧^#1	21″+1″[3M^∧^g^∧^#1]
TA7658	CS- *Ae. geniculata* DA 4M^∧^g^∧^#1	21″+1″[4M^∧^g^∧^#1]
TA7659	CS- *Ae. geniculata* DA 5M^∧^g^∧^#1	21″+1″[5M^∧^g^∧^#1]
TA7660	CS- *Ae. geniculata* DA 6M^∧^g^∧^#1	21″+1″[6M^∧^g^∧^#1]
TA7661	CS- *Ae. geniculata* DA 7M^∧^g^∧^#1	21″+1″[7M^∧^g^∧^#1]
TA2775	*Ae. peregrina*	U^p^U^p^S^p^S^p^
TA7594	CS- *Ae. peregrina* DA 1S^∧^v^∧^#1	21″+1″[1S^∧^v^∧^#1]
TA7595	CS- *Ae. peregrina* DA 2S^∧^v^∧^#1	21″+1″[2S^∧^v^∧^#1]
TA7596	CS- *Ae. peregrina* DA 3S^∧^v^∧^#1	21″+1″[3S^∧^v^∧^#1]
TA7597	CS- *Ae. peregrina* DA 4S^∧^v^∧^#1	21″+1″[4S^∧^v^∧^#1]
TA7598	CS- *Ae. peregrina* DA 5S^∧^v^∧^#1	21″+1″[5S^∧^v^∧^#1]
TA7600	CS- *Ae. peregrina* DA 7S^∧^v^∧^#1	21″+1″[7S^∧^v^∧^#1]
TA7614	CS- *Ae. peregrina* DA 1U^∧^v^∧^#1	21″+1″[1U^∧^v^∧^#1]
TA7615	CS- *Ae. peregrina* DA 2U^∧^v^∧^#1	21″+1″[2U^∧^v^∧^#1]
TA7616	CS- *Ae. peregrina* DA 3U^∧^v^∧^#1	21″+1″[3U^∧^v^∧^#1]
TA7617	CS- *Ae. peregrina* DA 4U^∧^v^∧^#1	21″+1″[4U^∧^v^∧^#1]
TA7618	CS- *Ae. peregrina* DA 5U^∧^v^∧^#1	21″+1″[5U^∧^v^∧^#1]
TA7619	CS- *Ae. peregrina* DA 6U^∧^v^∧^#1	21″+1″[6U^∧^v^∧^#1]
TA7620	CS- *Ae. peregrina* DA 7U^∧^v^∧^#1	21″+1″[7U^∧^v^∧^#1]
TA2780	*Ae. speltoides*	SS
TA7689	CS- *Ae. speltoides* DA 1S#3	21″+1″[1S#3]
TA7690	CS- *Ae. speltoides* DA 2S#3	21″+1″[2S#3]
TA7691	CS- *Ae. speltoides* DA 3S#3	21″+1″[3S#3]
TA7692	CS- *Ae. speltoides* DA 4S#3	21″+1″[4S#3]
TA7693	CS- *Ae. speltoides* DA 5S#3	21″+1″[5S#3]
TA7694	CS- *Ae. speltoides* DA 6S#3	21″+1″[6S#3]
TA7695	CS- *Ae. speltoides* DA 7S#3	21″+1″[7S#3]

### Development of STS markers based on the sequence of the barley *RPK* gene *HvRPK*

The sequence of the barley gene *HvRPK* from the barley BAC clone AF474072 was downloaded from the Genbank [16]. Two SSRs (simple sequence repeats) were identified within the *HvRPK* sequence using the SSRHUNTER [18], and several primers flanking the two SSR motifs were designed using Primer 3 Software (http://www.genome.wi.mit.Edu/cgi-bin/primer/primer3.cgi). The detailed information on all the primers used in this study is given in Table 2.

**Table 2:** Information of primers used in this study.

Primer name	Primer sequence (5′-3′)
RPK-F1/F2 as forward primers were combined with the reverse primers RPK-R1∼R4 and annealing temperatures were 65∼68°C.	
RPK-F1	CGACTACGTGACGCTCAAGA
RPK-F2	CAAGAGCCTCGACAAGATCC
RPK-R1	GCGAAGAGGATCTTGTCGAG
RPK-R2	ACTTGTCGTCGAGGAGGATG
RPK-R3	ACACCAGCATCACCTCCTTC
RPK-R4	AGGCGTCATCATCCAGTAGC
Primers were used to amplify the full length of *TaRPK1* and the annealing temperature was 61°C.	
TaRPK-ORF-F	ATGGCAGCCCGAGACACCAGTTCAA
TaRPK-ORF-R	TCATTTGCCGAAGCTCATGTCGTAG
Primers were used in the Q-RT-PCR analyses and the annealing temperature was 65°C.	
RPK-QPCR-F	TACTTCAGCGGCAACATGAG
RPK-QPCR-R	AAATCCTTGGTGGCCTTCTT
18S rRNA-F	AACACTTCACCGGACCATTCA
18S rRNA-R	CGTCCCTGCCCTTTGTACAC
Primers were used to amplify the ORF of *TaRPK1-2G* to construct the over-expression vector and the annealing temperature was 61°C.	
TaRPK1-2G-F	CGGGATCCATGGCAGCCCGAGACA
TaRPK1-2G-R	TCCCCCGGGTCATTTGCCGAAGCTC

### PCR amplification

For polymorphic analysis of the STS markers and chromosomal location of *TaRPK1*, PCR was performed following the procedure of Ji et al. (2008) [17]. The PCR products were separated in the 8% non-denaturing polyacrylamide gels (Acr: Bis = 19: 1 or 39: 1) at room temperature with 1×TBE buffer and visualized by silver staining [19]. High fidelity Taq DNA polymerases (QIAGEN, HotStar HiFidelity Polymerase Kit) were used for PCR amplifications for cloning of the gene *TaRPK1*. Amplification was performed at 94°C for 5 min; 32 cycles of 94°C for 15 s, 65 to 68°C for 1 min, and 72°C for 2 min; followed by 10 min at 72°C. PCR products were analyzed in 1% agarose gels.

### BAC library screening and *RPK* gene cloning in wheat

The BAC library of an elite common wheat variety Xiaoyan 54, kindly provided by Dr. Hongqing Ling, Institute of Genetics and Development Biology Chinese Academy of Sciences, China, was used to isolate the *TaRPK* gene. The positive BAC clone was identified by PCR using a STS marker STS_RPK-F1/R4_ specific to the *RPK* sequence following the procedure of Dong et al. [20]. *HvRPK* was located in a 4.3 kb fragment when the BAC clone AF474072 was digested by *Xba* I [16]. To sub-clone the *TaRPK* from the positive BAC clone of Xiaoyan 54, the BAC was also digested with *Xba* I and the expected full-length DNA sequence of *TaRPK* gene was obtained. Based on the *TaRPK* sequence obtained from Xiaoyan 54, PCR primers flanking the ORF of *TaRPK* were designed and used to clone the full-length genomic DNA and cDNA sequences of the *TaRPK1* gene from IGVI-465 and *T. timopheevii*.

### Splicing feature, protein architecture and phylogenetic analyses

To characterize the splicing feature of the gene *TaRPK1-2G*, the genomic DNA and cDNA sequences of *TaRPK1-2G* were used for alignment and similarity analysis by DNAMAN version 5.2.2. TMHMM (http://www.cbs.dtu.dk/services/TMHMM-2.0/) [21] and SMART (http://smart.embl-heidelberg.de/) [22] were used to identify the architectures of the protein domain.

The kinase domain of TaRPK1-2G was used as query sequence for identifying the TaRPK1-2G orthologs by the BLASTP searches in the Phytozome proteome database (http://www.phytozome.net/search.php?show=blast&method=Org_Cpapaya), and the best hits in various plant species were used for further analysis when the amino acid identity was >50% identical (e-values<e^−60^) for at least 100 amino acid of the query sequence. The amino acid sequences of identified orthologs in fasta format were retrieved from the Phytozome proteome database, and the kinase domains of these orthologs were identified by the SMART protein domain prediction server [22]. The kinase domain sequences of TaRPK1-2G and its orthologs were aligned using the CLUSTALX [23]. Phylogenetic analysis was conducted and viewed using MEGA version 4.1 [24] based on the Neighbor–Joining method with a Poisson correction model and a bootstrap test of 1000 replicates.

### Expression analysis of *TaRPK1-2G* by quantitative RT-PCR

Seedlings of the ‘IGVI-465’ were grown in a growth chamber at 16/8 h light/dark and 25/20°C temperature regime until the second leaf stage, when inoculation of *Blumeria graminis* f.sp. *tritici* (*Bgt*) was conducted by dusting the conidia from a susceptible wheat variety Sumai 3 onto the surface of the leaves of ‘IGVI-465’. The source of *Bgt* inoculum was a local natural mixture collected in the wheat fields of Nanjing, Jiangsu province, China. For chemical treatments, the seedlings of ‘IGVI-465’ at the first leaf stage were sprayed with 5 mM salicylic acid (SA), 100 µM methyl jasmonate (MeJA), 100 µM abscisic acid (ABA), and 7 mM H_2_O_2_, respectively. Total RNA of each sample was extracted using the TRIZOL reagent (Invitrogen, USA), and 2 µg of RNA per sample was used for synthesizing the first-strand cDNA by the AMV reverse transcriptase (Takara) following the manufacturer's instruction.

Responses of the *TaRPK1-2G* upon infection with *Bgt* and the application of chemical agents were analyzed by the real-time quantitative RT-PCR (Q-RT-PCR) with the specific primer pair of *TaRPK1-2G* (RPK-QPCR-F and RPK-QPCR-R), and the 18S rRNA (amplified with primers 18rRNA-F and 18S rRNA-R) was used as a control. The PCR reaction was performed in a 25 µl reaction mixture containing 1×SYB Premix Ex Taq (Takara), 0.2 µM of each primer, 1× Rox Reference Dye, and about 30 ng cDNA per sample using the ABI Prism 7000 system (Applied Biosystems, USA). The program used was as follows: 1 min at 94°C; followed by 40 cycles of 5 s at 94°C, 15 s at 68°C and 30 s at 72°C. After completion of the reactions, the cycle threshold (C_T_) value was calculated and subsequently, the ΔΔC_T_ algorithm was used [25]. The final value of relative gene quantification was expressed as *n*-fold change in *TaRPK1-2G* transcript level in tested samples in comparison with the untreated controls. The results are presented as the mean ± standard deviation (SD) of three independent analyses.

### Single-cell transient expression assay

The ORF of *TaRPK1-2G*, amplified using the primer pair TaRPK1-2G-F and TaRPK1-2G-R, was inserted into the expression vector pBI220 by placing the *TaRPK1-2G* under the control of the CaMV35S promoter. The recombinant vector pBI220-*TaRPK1-2G* was used for single-cell transient expression assay according to Shirasu et al. [26] and Cao et al. [14]. The reporter plasmid pAHC25 containing the β-glucuronidase (*GUS*) gene and the expression plasmid pBI220-*TaRPK1-2G* were mixed before coating of the particles (molar ratio of 1∶1; 1 µg of total DNA). The bombarded leaves were transferred to 1% agar plates supplemented with 85 µM benzimidazole and incubated at 18°C for 8 h before high density inoculation with *Bgt* spores. Leaves were stained for GUS to identify the *TaRPK1-2G* transformed cells at 48 h after *Bgt* inoculation. The haustorium index (percentage of GUS-staining cells with haustoria in the total GUS-staining cells attacked by *Bgt*) was computed based on mean of three independent experiments, each based at least 50 independent interaction events.

### Statistical analysis

For the results of Q-RT-PCR and single-cell transient expression assay, the obtained data were analyzed by using SPSS analytical software package (version 19.0, IBM Corporation, New York, USA), and presented as the mean ± standard deviation (SD) of three independent replicates (*n* = 3). Comparisons between the controls and the tested samples were made ANOVA test and the statistical differences between the groups were tested by the Duncan's test. All statistically significant differences were tested at the level of *P*<0.05. Figures were drawn by Origin Data Analysis and Graphing Software, OriginPro8 (Version8.5.1, OriginLab Corporation, Massachusetts, USA).

## Results

### Development of *RPK* gene-based STS markers and analysis of their potential use as functional marker in wheat and other *Triticeae* species

**Figure 1:**
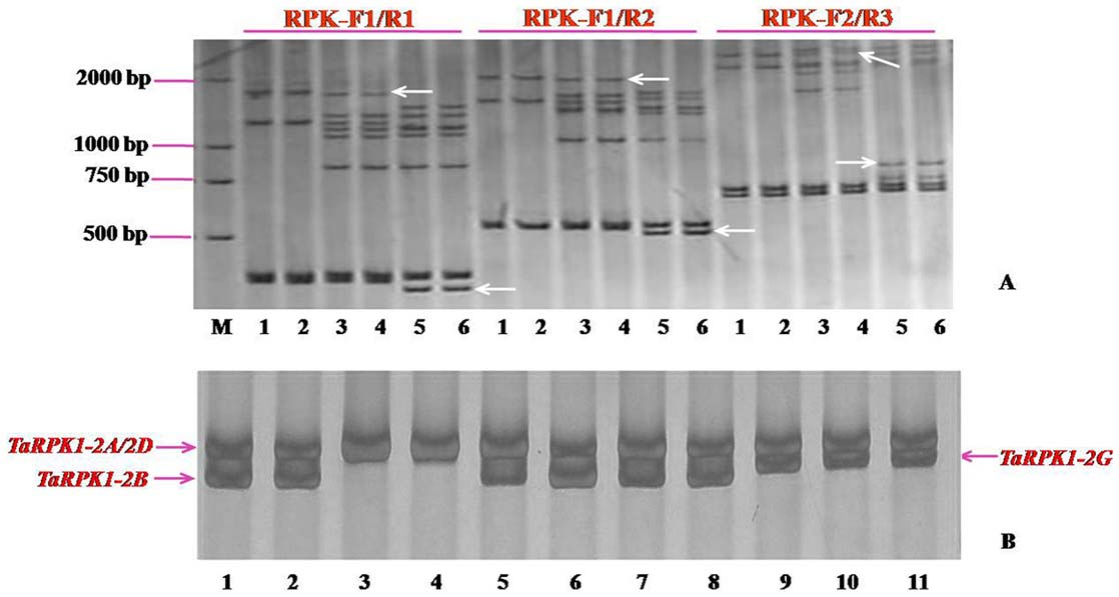
Polymorphic analysis of *HvRPK*-based STS primer pair amplicons and chromosome location of the *RPK* amplicons in common wheat. A. Polymorphisms between the *Pm6*-carrying powdery mildew resistant lines and susceptible lines (Arrows indicate the polymorphic bands). M: DNA fragment marker DL2000; 1 and 2: *Triticum timopheevii* (with *Pm6*); 3 and 4: *Pm6*-carrying *T. aestivum*-*T. timopheevii* introgression line IGVI-465; 5 and 6: susceptible wheat variety Prins. B. Chromosome location of the *RPK* gene using the homoeologous group-2 ‘Chinese spring’ (CS) nulli-tetrasomic (NT) lines and *Pm6*-carrying *T. aestivum*-*T. timopheevii* introgression lines. The left arrows show the specific bands from chromosomes 2A or 2D and 2B, and arrow on the right shows the specific band from the chromosome 2G of *Pm6*-carrying lines. 1: Nulli2A/Tetra2B; 2: Nulli2A/Tetra2D; 3: Nulli2B/Tetra2A; 4: Nulli2B/Tetra2D; 5: Nulli2D/Tetra 2A; 6: Nulli2D/Tetra2B; 7: CS; 8: Prins; 9: *Pm6*-carrying *T. aestivum*-*T. timopheevii* introgression line IGVI-465; 10: *Pm6*-carrying *T. aestivum*-*T. timopheevii* introgression line IGVI-466; 11: *T. timopheevii* (with *Pm6*).

The barley *RPK* gene (*HvRPK*) and the RFLP marker BCD135, which closely linked with gene *Pm6* in wheat, are both present in the barley BAC clone AF474072. To detect whether the orthologs of *HvRPK* was also linked with *Pm6* in wheat, two forward (RPK-F1 and RPK-F2) and four reverse (RPK-R1, RPK-R2, RPK-R3, and RPK-R4) primers, which flanked the SSR motifs of the *HvRPK* gene, were designed. Eight primer pairs were combined and used to test the polymorphism between the *Pm6*-carrying lines and their susceptible recurrent parent ‘Prins’. It was found that four primer combinations, STS_RPK-F1/R1_, STS_RPK-F1/R2_, STS_RPK-F2/R3_ and STS_RPK-F1/R4_ could produce clear polymorphism (Figure 1A and B). They produced distinct specific bands for both the *Pm6*-carrying lines and ‘Prins’, indicating they could serve as co-dominant markers. Linkage analysis in a previous study has shown that the wheat *RPK* gene is closely linked to the *Pm6* [27].

**Figure 2:**
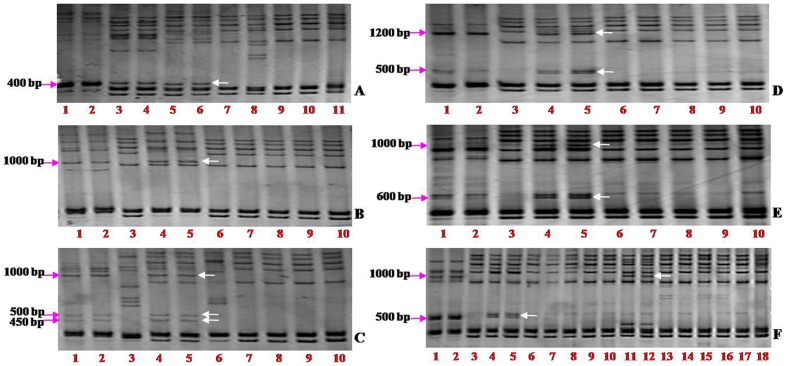
Conserved *RPK* genes identified by the STS marker STS_RPK-F1/R1_ from the homoeologous group-2 chromosomes of a series of wheat and *Triticeae* species. Arrows indicate the *RPKs* specific to each species. A: Barley and ‘Chinese spring’ (CS)-barley disomic chromosome addition (DA) lines. Lanes 1 and 2: Barley variety BETZES; 3 and 4: DA2H; 5 and 6: DAt”[2H] (ditelosomic chromosome addition line); 7: DA3H; 8: DA4H; 9: DA5H; 10: DA6H; 11: DA7H. B: Rye and CS-rye DA lines. Lanes 1 and 2: Rye variety BLANCO; 3: DA1R; 4 and 5: DA2R; 6: DA3R; 7: DA4R; 8: DA5R; 9: DA6R; 10: DA7R. C. *Ae. speltoides* and CS-*Ae. Speltoides* DA lines. Lanes 1 and 2: *Ae. speltoides*; 3: DA1S; 4 and 5: DA2S; 6: DA3S; 7: DA4S; 8: DA5S; 9: DA6S; 10: DA7S. D. *Ae. longissima* and CS-*Ae. longissima* DA lines. Lanes 1 and 2: *Ae. longissima*; 3: DA1S^l^; 4: DA2S^l^#3-1; 5: DA2S^l^#3-2; 6: DA3S^l^ ; 7: DA4S^l^; 8:DA5S^l^; 9: DA6S^l^; 10: DA7/4S^l^. E. *Ae. geniculata* and CS-*Ae. geniculata* DA lines. Lanes 1 and 2: *Ae. geniculata*; 3: DA1M^g^; 4 and 5: DA2M^g^; 6: DA3M^g^; 7: DA4M^g^; 8: DA5M^g^; 9: DA6M^g^; 10: DA7M^g^. F: *Ae. peregrina* and CS-*Ae. peregrina* DA lines. Lanes 1 and 2: *Ae. peregrina*; 3: DA1S^p^; 4 and 5: DA2S^p^; 6: DA3S^p^; 7: DA4S^p^; 8: DA5S^p^; 9: DA7S^p^; 10: DA1U^p^; 11 and 12: DA2U^p^; 13: DA3U^p^; 14: DA4U^p^; 15: DA5U^p^; 16: DA6U^p^; 17: DA7U^p^; 18: CS.

The amplification of STS_RPK-F1/R4_ in the Chinese Spring (CS) nulli-tetrasomic lines involving homoeologous group-2 chromosomes enabled the clear discrimination of the *RPK* genes on the chromosomes 2G and 2B, while the two *RPK* gene copies on chromosomes 2A and 2D could not be discriminated due to their similar size (Figure 1B). The presence of *RPKs* in the 2A, 2B and 2D indicated their conservation in the three wheat genomes. To further demonstrate the conservation of the *RPK* gene in homoeologous group-2 chromosomes in the genomes of wheat relatives, the *RPK* genes were amplified using the above four STS markers in wheat relatives and their corresponding alien addition lines in the background of Chinese Spring. The results showed that all these STS markers could produce specific fragments in the alien species and the addition lines involving the group-2 chromosomes. For example, STS_RPK-F1/R1_ produced a ∼1900 bp specific band from chromosome 2G of *T. timopheevii* (Figure 1B), a ∼400 bp specific band from chromosome 2H of barley (Figure 2A), a ∼1000 bp specific band from 2R of rye (Figure 2B), three specific bands (∼480 bp, ∼500 bp, and ∼1000 bp) from 2S of *Ae. speltoides* (Fig. 2C), two specific bands (∼1200 bp and ∼500 bp) from 2S^l^ of *Ae. longissima* (Figure 2D), two specific bands (∼1000 bp and ∼600 bp) from 2M^g^ of *Ae. geniculata* (Figure 2E), a ∼500 bp and a ∼1000 bp specific band from 2S^p^ and 2U^p^ of *Ae. peregrina*, respectively (Figure 2F). STS_RPK-F1/R1_ could clearly discriminate the chromosomes 2S^p^ and 2U^p^ in the tetraploid *Ae. peregrina*. All these indicated highly conservation of *RPK* genes among diverse species, and the STS markers derived from *RPK* gene can be served as conserved functional markers to identify specific homoeologous group-2 chromosomes or chromosome segments of wheat relatives.

### Isolation of *TaRPK* gene in the common wheat variety Xiaoyan 54

The fragment amplified by the STS_RPK-F1/R4_ only included a 1,032 bp region of the CDS of barley *HvRPK*, which was totally 1,281 bp in length. To obtain the full length of the *HvRPK* homologs in hexaploid wheat, STS_RPK-F1/R4_ was used to screen the BAC library constructed from the wheat variety Xiaoyan 54. Ten positive clones were identified. Based on the digestion results of the BAC clone AF474072 using *Xba* I [16], the *HvRPK* was located in a 4.3 kb digested fragment. Accordingly, the positive clones were digested by *Xba* I, and an expected ∼4.3 kb target fragment was sub-cloned into the pBluescript vector for sequencing. It was found that this 4,305 bp fragment (BAC-xiaoyan54, GenBank accession number JX065225) included two putative genes with opposite transcript directions, one was a putative PLA IIB/PLP6 (Patatin-like protein 6) gene of 1,166 bp in length, and the other was the target *RPK* gene of 1,644 bp in length. We designated the two genes from wheat as *TaPLP6* and *TaRPK*, respectively.

### Cloning of *TaRPK* homologs from different genomes, analysis of its splicing feature and putative protein domain architectures in the *Pm6*-carrying line IGVI-465

As *TaRPK* was tightly linked with *Pm6* [27], and mapped to the introgression segment of 2G. It is interesting to isolate the *TaRPK* homologous gene from the *Pm6*-carrying line IGVI-465 and to figure out its putative biological function. Based on the sequence of *TaRPK* from Xiaoyan 54, PCR primers (TaRPK-ORF-F and TaRPK-ORF-R) flanking the ORF (open reading frame) of *TaRPK* were used to clone the full-length genomic DNA and cDNA sequences of the *TaRPK* homologs from IGVI-465 and *T. timopheevii*. Sequence analysis indicated that three *TaRPK* homologs were present in IGVI-465 on chromosomes 2A (*TaRPK1-2A*, 1,691 bp, GenBank accession number JX065226), 2D (*TaRPK1-2D*, 1,692 bp, GenBank accession number JX065228), and the introgressed chromosome segment of 2G (*TaRPK1-2G*, 1,650 bp, GenBank accession number JX065231). Two *TaRPK* homologs were present in *T. timopheevii*, one each on chromosomes 2A^t^ (*TaRPK1-2A^t^*, 1,692 bp, GenBank accession number JX065227) and 2G (*TaRPK1-2G*, 1,650 bp). This indicated that in wheat variety CS, there were also three copies of *RPK*, and the two copies from chromosomes 2A and 2D in both CS and IGVI-465 were hard to separate and visualized as a single band in the non-denaturing polyacrylamide gels (as shown in Figure 1B) due to their similar sequence size (with only one base pair difference in size as shown in IGVI-465). The 100% identity of the two *TaRPK1-2G* from IGVI-465 and *T. timopheevii* further proved that *TaRPK1-2G* was located on the introgressed chromosomal fragment of 2G in IGVI-465. Multiple sequence alignment showed that the *TaRPK* from Xiaoyan 54 and *TaRPK1-2A* had the highest similarity (being 93.27% identity) indicating that *TaRPK* was originally from the chromosome 2A in Xiaoyan 54.

**Figure 3:**
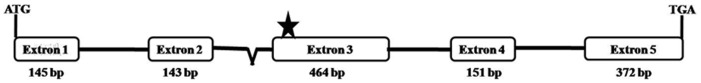
Schematic diagram of the *TaRPK1-2G* gene and its alternative splicing feature. Exons are shown as boxes. Introns are shown as black lines. The alternative splicing site is shown as diagonal lines. The star represents the premature stop codons in the alternative transcript.

The comparison of the genomic DNA of the three *TaRPK1* homologs with their corresponding cDNA sequence in IGVI-465 showed that *TaRPK1-2G* was the gene actively transcribed under the tested conditions. *TaRPK1-2G* produced two different transcripts, probably due to alternative splicing (Figure 3). One type (*TaRPK1-2G -1*, GenBank accession number JX065229) had the complete ORF and generated the putative functional protein, whereas the other (*TaRPK1-2G-2*, GenBank accession number JX065230) had 13 bp intronic sequence from the second intron, and resulted in a truncated protein due to premature termination (Figure 3, indicated by the star). Alignment of the *TaRPK1-2G-1* and *TaRPK1-2G* indicated that *TaRPK1-2G* had 4 introns and 5 extrons (Figure 3), and *TaRPK1-2G -1* coded for a predicted protein of 424 amino acids. Based on structural properties indicated by the TMHMM [21] and SMART programs [22], the predicted protein was a receptor protein kinase (RPK). The predicted protein TaRPK1-2G has a transmembrane domain (TM, from the 7^th^ to 29^th^ amino acid), an N-terminal hydrophobic signal peptide (from the 1^st^ to 26^th^ amino acid), and a cytoplasmic serine/threonine protein kinase domain (from the 80^th^ to 358^th^ amino acid), but does not have the typical extracellular structure such as eLRRs. We found that, in the transmembrane domain and the signal peptide, there were 20 amino acids overlapping, which has not been found in the *Arabidopsis* RLKs.

### Analysis of TaRPK1-2G orthologs in plant species

**Figure 4:**
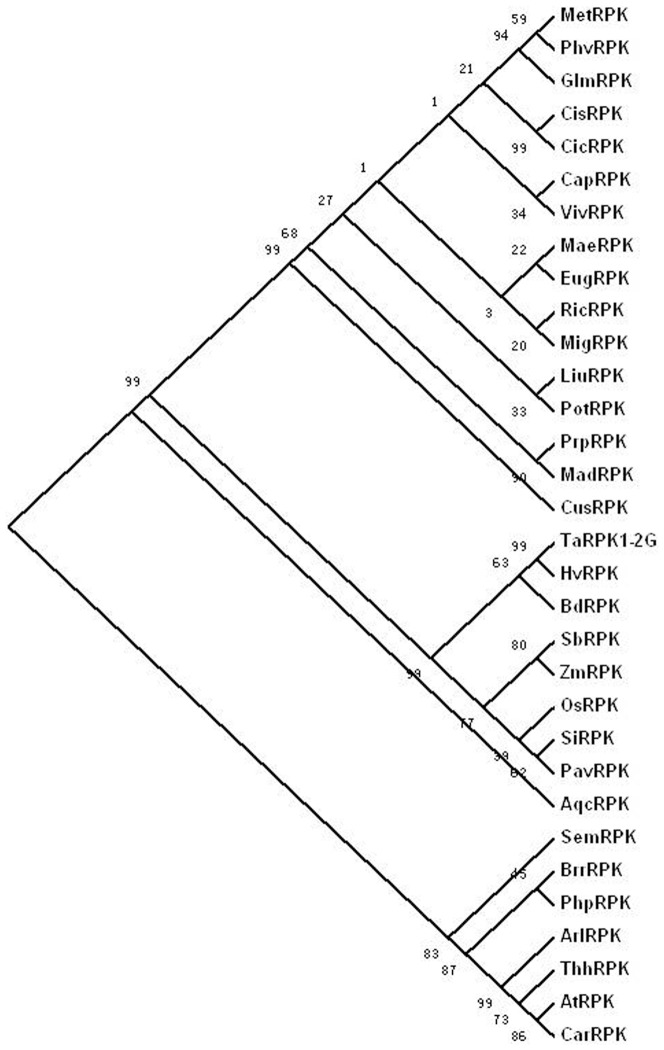
Phylogenetic tree of TaRPK1-2G and its orthologs. Neighbor Joining phylogenetic tree of the kinase domains of TaRPK1-2G and its orthologs from other 31 plant species was constructed using MEGA4.1. Bootstrap support on the left of each node was inferred from 1,000 replicates. The full names of all species and accession number or locus name of each sequence used for phylogenetic analysis were provided in Form S1.

To reveal the relationship of TaRPK1-2G with other RPKs, BLASTP was performed against the Phytozome proteome database for identifying TaRPK1-2G orthologs based on the similarity in their kinase domains, which was considered to be the most conserved domain among the RPKs. Orthologs were identified from 30 plant species with available genomic information, and together with the barley HvRPK, a total of 31 orthologs were used for phylogenetic analysis. These orthologs share a conserved serine/threonine protein kinase domain and belong to serine/threonine protein kinase by KOG classification (Form S1). The results indicated that TaRPK1-2G was highly conserved among the 32 selected species, and the amino acid sequence identity in the kinase domain was more than 86% among the RPKs from various grass species, including wheat (TaRPK1-2G), barley (HvRPK), *Oryza sativa* (OsRPK), *Brachypodium distachyon* (BdRPK), *Sorghum bicolor* (SbRPK), *Setaria italica* (SiRPK), *Zea mays* (ZmRPK), and *Panicum virgatum* (PavRPK) (Figure 4, detail information on these orthologs were provided in Form S1). Comparative genome mapping of the *TaRPK1-2G* orthologs in grass species showed that they were well retained in the collinear regions of *TaRPK1-2G* which have been revealed by whole-genome comparison [28], [29], such as *TaRPK1-2G* and *Pm6* co-located on 2BL [fraction length (FL) 0.50–1.00 [27], *HvRPK* on chromosome 2H: 140.1 cM, *OsRPK* on Chr4: 33,594,296 bp–33,596,099 bp, *BdRPK* on Bd5: 26,167,244 bp–26,168,882 bp, and *SbRPK* on chromosome_6: 59,793,399 bp–59,795,140 bp (The locations of these *RPKs* in their corresponding genomes are shown in Form S1). Moreover, these orthologs from the grass species formed into a group, which was separate from the dicots, with a high bootstrap support value (99%). However, TaRPK1-2G was most similar (with 97% identity) to the putative HvRPK from the barley BAC clone AF474072 (AAM13439). In *Arabidopsis*, the putative LRR-RLK (AT1G56140) showed the highest identity (53.8%) with TaRPK1-2G. AT1G56140 has been classified as the LRR protein of the VIII-2 subfamily [4], suggesting that TaRPK1-2G, although without the LRR motifs at its extracellular structure, also belongs to the VIII-2 subfamily (Figure 4).

### Expression profiling of *TaRPK1-2G* under stress and hormone treatments

**Figure 5:**
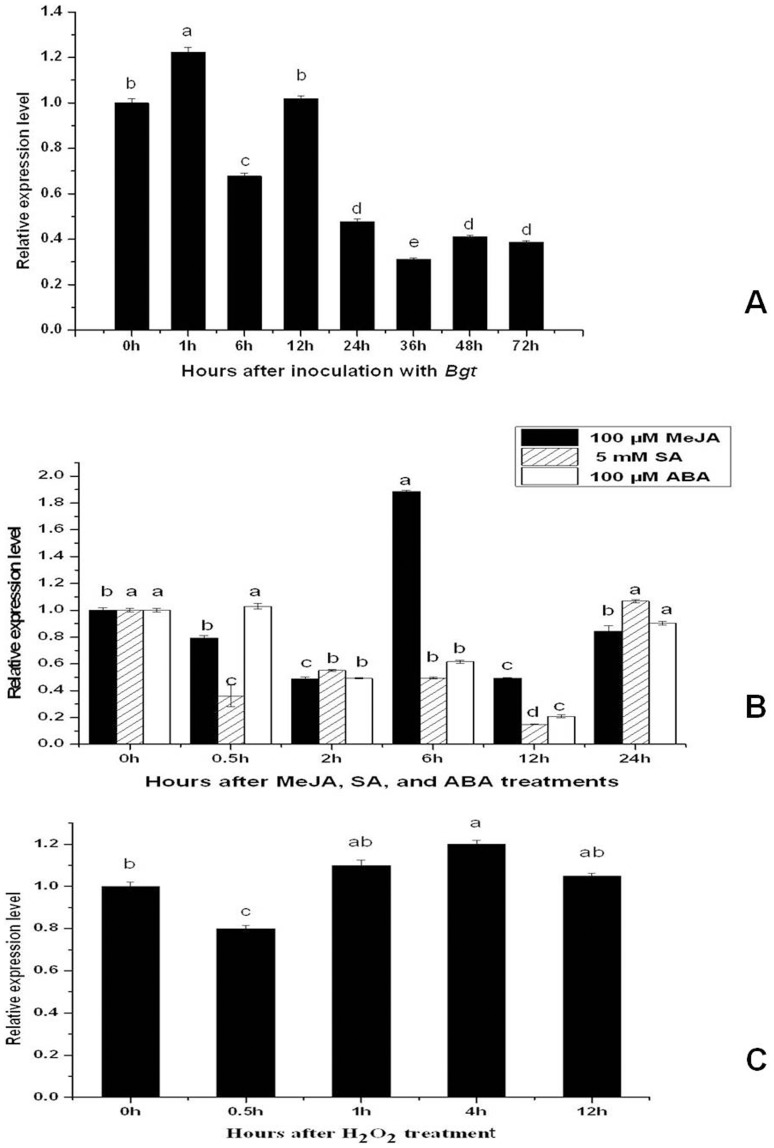
Expression of *TaRPK1-2G* in the leaves of IGVI-465. (A) inoculated with *Bgt*. (B) treated with 100 µM MeJA, 5 mM SA and 100 µM ABA. (C) treated with 7 mM H_2_O_2_. Bars with different letters show significant differences at the *P*<0.05 level.

Since a large number of *RPKs* were proved to participate in plant defense response. To investigate whether *TaRPK1-2G* also participates in the defense response in wheat, the expression patterns of *TaRPK1-2G* upon the attack of *Bgt* infection and different hormone applications were analyzed by Q-RT-PCR. It was found that expression of *TaRPK1-2G* was up-regulated by the *Bgt* infection in the *Pm6*-containing line IGVI-465 at 1 h after inoculation (hai), but then reduced its expression at 6 hai and maintained at a low level from 24 hai to 72 hai (Figure 5A), suggesting that *TaRPK1-2G* might take part in the active defense response to *Bgt*. Expression of *TaRPK1-2G* was significantly induced by MeJA, but down-regulated by SA and ABA (Figure 5B). Application of H_2_O_2_ also regulated the *TaRPK1-2G* expression, although not dramatically (Figure 5C). The induction of *TaRPK1-2G* transcription by *Bgt* and MeJA treatments in IGVI-465 suggested that this gene played a role in defense response to the infection by *Bgt* or other biotic or abiotic stresses via the JA pathway.

### *TaRPK1-2G*-mediated resistance to *Bgt* revealed by single-cell transient expression assay

**Figure 6:**
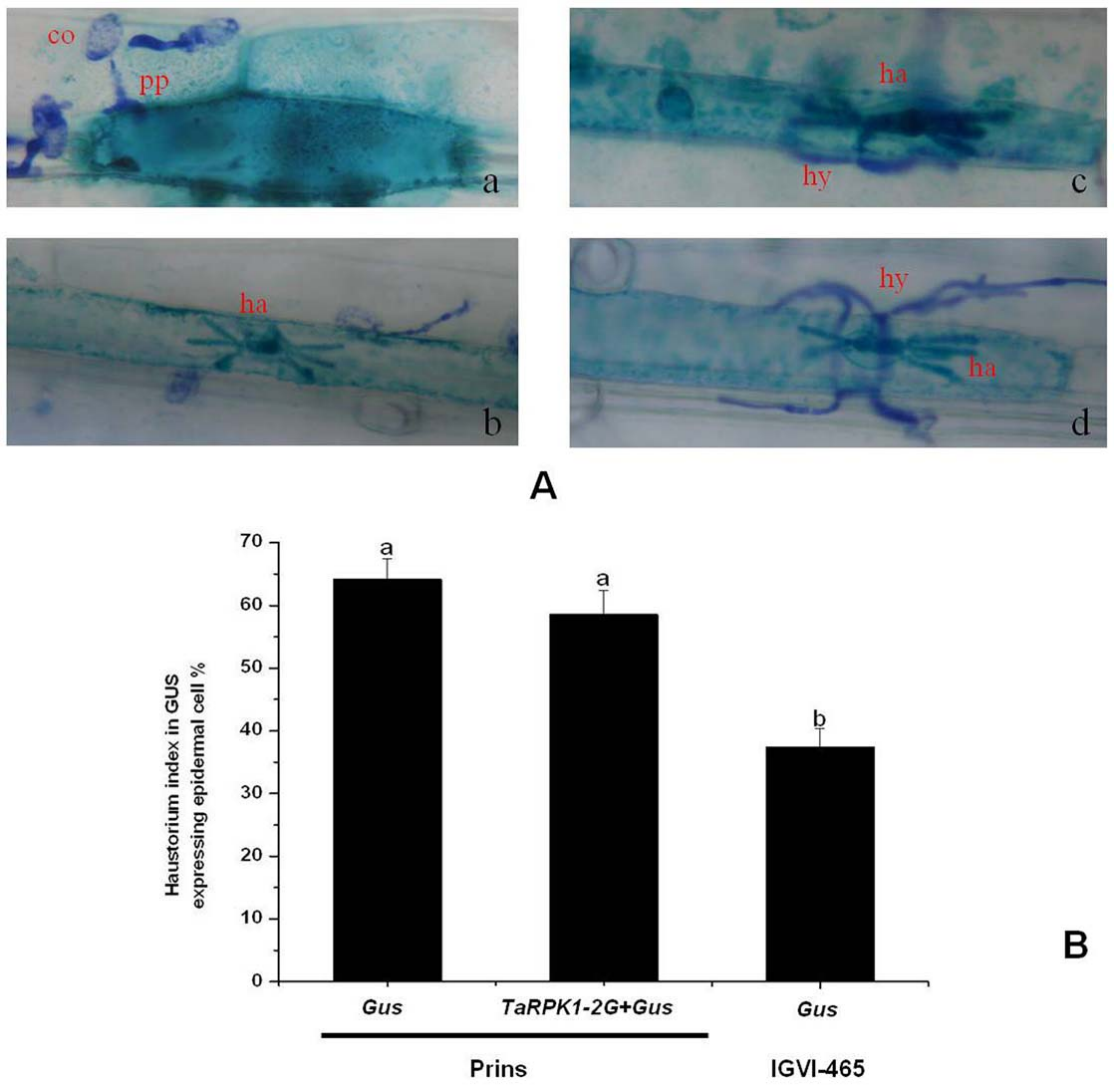
Functional analysis of the *TaRPK1-2G* by single cell transient expression assay in interaction with *Bgt*. A. The compatible and incompatible interactions between the positive transformed cells of the target gene with *Bgt* fungus. a: incompatible interaction in which the formation of haustorium failed. b, c, d: compatible interaction in which the formation of haustorium succeeded. co: conidia; pp: penetration peg; ha: haustorium; hy: hyphae. B. The haustorium index in the *GUS* transformed cells or in the *GUS* and *TaRPK1-2G* co-transformed cells. Bars with different letters show significant differences at the *P*<0.05 level; differences of the haustorium index were compared between the *GUS* transformed cells and the *TaRPK1-2G*+*GUS* co-transformed cells using Prins (susceptible) as the receptors, and between the *GUS* transformed cells using IGVI-465 (resistant) as the receptor.

The localization of *TaRPK1-2G* to the same chromosome region of *Pm6* on the chromosome 2G fragment and its up-regulation by *Bgt* infection hinted the possible correlation of *TaRPK1-2G* with powdery mildew resistance. This assumption was tested using a single-cell transient expression assay. Single-cell transient expression assay is highly efficient in assessing the function of defense-related genes in response to *Bgt* both in wheat and barley, especially for those genes with pre-haustorium resistance. Haustorium is a key structure for nutrient extraction during *Bgt* development. The haustorium index (HI) can be used as a criterion to estimate the compatibility of interaction between the host and *Bgt*. In the single-cell transient assay, the epidermal cells expressing *GUS* gene and challenged by the *Bgt* were selected as the targets. If *Bgt* failed to penetrate into the cell and no haustorium formed, the interaction was considered to be incompatible (Figure 6A-a); on the other hand, if the haustorium and elongating secondary hyphae were observed, the interaction was considered to be compatible (Figure 6A-b, c, d). In the susceptible variety Prins, the HI was 64.16% when transformed with the *GUS* gene alone, and 59.53% when co-transformed with *GUS* and *TaRPK1-2G*. In the resistant line IGVI-465, the HI was 37.47% when transformed with the *GUS* gene (Figure 6B). Statistical analysis indicated that there was no significant difference for the HI between the *GUS* alone transformation and the *TaRPK1-2G*+*GUS* co-transformation in the susceptible variety Prins, while significant difference was observed for the HI between the *GUS* alone transformation in Prins and IGVI-465. These indicated that transciently over-expression of *TaRPK1-2G* could prevent the formation of haustoria to a certain extent, but it could not improve the resistance level of the susceptible variety to *Bgt*. We deduced that the *TaRPK1-2G* play a role in the powdery mildew resistance pathway of IGVI-465, but it should not be the best candidate for the *Pm6* gene.

## Discussion

In this study, a conserved wheat *RPK* gene associated with the introgressed *Pm6* from *T. timopheevii* was cloned and characterized. Based on the *HvRPK* gene, STS markers were developed and they could be used to identify the alien chromosomes belonging to the homoeologous group-2 in the wheat background. Molecular mapping and bioinformatics analysis revealed orthologous copies of *RPK* in the *Triticeae* and apparent orthologs were present in various plant species. The orthologs of *RPK* were conserved in the collinear regions of rice, *Brachypodium*, *S. bicolor*, and *Z. mays* (The locations of these *RPKs* in their corresponding genomes are shown in Form S1), and the collinear regions of *TaRPK1-2G* orthologs in these grass species have been revealed by whole-genome comparison [28], [29]. This implied that the conserved *RPK* gene may confer important biological roles in plant species.

The presence of a putative hydrophobic signal peptide, a hydrophobic membrane-spanning segment, and a highly conserved kinase domain supported that TaRPK1-2G is a receptor like protein kinase. Although the kinase domain-based multiple sequence alignment and phylogenetic analysis supported the TaRPK1-2G could be classified into the LRR VIII-2 subfamily, the fact that it lacks of the LRR motifs at the extracellular structure implied that TaRPK1-2G may be a new member of the LRR VIII-2 subfamily. Among those genes closely related to TaRPK1-2G, none of their function has been elucidated. *TaRPK1-2G* was found to be up-regulated by both *Bgt* and MeJA treatments, suggesting that it might be involved in the defense response. However, the exact function of *TaRPK1-2G* and its orthologs in the grass species remains to be further elucidated.

Alternative splicing of pre-mRNA is a prominent post-transcriptional mechanism, which contributes to increased protein diversity and the regulation of gene function in eukaryotic cells [30], [31]. It was estimated that about 40–60% genes in human, at least 42% in *A. thaliana* and 56% in maize undergo alternative splicing [32]–[34]. The impact of the alternative splicing has been frequently reported in human, which sometimes leads to many diseases [35]–[38]. In plants, alternative splicing was reported to partake in the regulation of flowering, disease resistance, stress tolerance, grain quality and so on [39]. For example, the photoperiod gene *Ppd-B1*, which plays a major role in controlling of the heading time, yield, and adaptability in wheat, was alternatively spliced [40]. The disease resistance gene *RPS4* in *Arabidopsis* produces multiple transcripts via alternative splicing, and *RPS4*-mediated resistance requires the combined presence of transcripts encoding both the full-length and the truncated ORFs [41]. In wheat, the transcription factor *DREB2* in response to different abiotic stresses can produce three types of transcripts in varying amounts through alternative splicing. Alternative splicing of *RPS4* undergoes dynamic changes specifically during the resistance response [42], and alternative splicing of *DREB2* was regulated by the ABA-dependent (response to drought and salt stress) and ABA independent pathway (response to low temperature stress) [43]. These findings indicate the importance of alternative splicing in regulation of responses to various stresses. In this study, we found that *TaRPK1-2G* was also an alternative splicing gene producing two different transcripts, *TaRPK1-2G-1 and TaRPK1-2G-2*, however, only the *TaRPK1-2G-1* encoded the full-length ORF, and it was mainly up-regulated by the MeJA. The relationship between the alternative splicing of *TaRPK1-2G* and JA signaling defense pathway is still unknown, and it will be important to characterize the function of *TaRPK1-2G* and its regulation at the post-transcriptional level.

## Supporting Information

Form S1 **The information of TaRPK1-2G orthologs.** The kinase domain of TaRPK1-2G was used as query sequence for identifying the TaRPK1-2G orthologs by BLASTP searches in the Phytozome proteome database. The best hits in each species were retrieved. This form provided the information about the species of the orthologs, accession number & locus name, location in corresponding genome, description of putative protein, and scores, e-values, identities, and positives of the BLAST results.
(XLS)
